# Promoting transportation safety in adolescence: the drivingly randomized controlled trial

**DOI:** 10.1186/s12889-023-16801-6

**Published:** 2023-10-17

**Authors:** Jessica Hafetz, Catherine C. McDonald, D. Leann Long, Carol A. Ford, Thandwa Mdluli, Andrew Weiss, Jackson Felkins, Nicole Wilson, Bradley MacDonald

**Affiliations:** 1 Department of Clinical and Health Psychology, Centre for Applied Developmental Psychology, The University of Edinburgh, Edinburgh, UK; 2 Penn Injury Science Center, University of Pennsylvania School of Nursing, Philadelphia, USA; 3 School of Public Health, Department of Biostatistics, University of Alabama at Birmingham, Birmingham, USA; 4 The Children’s Hospital of Philadelphia, University of Pennsylvania School of Medicine, Philadelphia, USA; 5 University of Pennsylvania School of Nursing, Philadelphia, USA; 6 The University of Edinburgh, Department of Clinical and Health Psychology, Edinburgh, UK

**Keywords:** Teen drivers, Motor vehicle crashes, Teen driver safety, Injury prevention, Driver training

## Abstract

**Background:**

The impact of young drivers’ motor vehicle crashes (MVC) is substantial, with young drivers constituting only 14% of the US population, but contributing to 30% of all fatal and nonfatal injuries due to MVCs and 35% ($25 billion) of the all medical and lost productivity costs. The current best-practice policy approach, Graduated Driver Licensing (GDL) programs, are effective primarily by delaying licensure and restricting crash opportunity. There is a critical need for interventions that target families to complement GDL. Consequently, we will determine if a comprehensive parent-teen intervention, the Drivingly Program, reduces teens’ risk for a police-reported MVC in the first 12 months of licensure. Drivingly is based on strong preliminary data and targets multiple risk and protective factors by delivering intervention content to teens, and their parents, at the learner and early independent licensing phases.

**Methods:**

Eligible participants are aged 16-17.33 years of age, have a learner’s permit in Pennsylvania, have practiced no more than 10 h, and have at least one parent/caregiver supervising. Participants are recruited from the general community and through the Children’s Hospital of Philadelphia’s Recruitment Enhancement Core. Teen-parent dyads are randomized 1:1 to Drivingly or usual practice control group. Drivingly participants receive access to an online curriculum which has 16 lessons for parents and 13 for teens and an online logbook; website usage is tracked. Parents receive two, brief, psychoeducational sessions with a trained health coach and teens receive an on-road driving intervention and feedback session after 4.5 months in the study and access to DriverZed, the AAA Foundation’s online hazard training program. Teens complete surveys at baseline, 3 months post-baseline, at licensure, 3months post-licensure, 6 months post-licensure, and 12 months post-licensure. Parents complete surveys at baseline, 3 months post-baseline, and at teen licensure. The primary end-point is police-reported MVCs within the first 12 months of licensure; crash data are provided by the Pennsylvania Department of Transportation.

**Discussion:**

Most evaluations of teen driver safety programs have significant methodological limitations including lack of random assignment, insufficient statistical power, and reliance on self-reported MVCs instead of police reports. Results will identify pragmatic and sustainable solutions for MVC prevention in adolescence.

**Trial Registration:**

ClinicalTrials.gov # NCT03639753.

**Supplementary Information:**

The online version contains supplementary material available at 10.1186/s12889-023-16801-6.

## Background

The long-term goal of this research is to identify effective programs that can reduce adolescents’ risk for motor vehicle crashes (MVCs). The impact of teen and young adult MVCs is substantial, with young drivers constituting only 14% of the US population but contributing to 30% of all fatal and nonfatal injuries due to MVCs and 35% ($25 billion) of associated medical and lost productivity costs [1]. The current best-practice policy approach to teen driver MVC prevention, Graduated Driver Licensing (GDL) programs, have been effective largely by delaying licensure and restricting crash opportunity; they have not, however, directly led to increases in young drivers’ skills or competencies [2, 3]. In other words, GDL’s success has come from its focus on restricting *access* to high risk contexts and not on directly improving drivers’ *competence*. There is a critical unmet need for efficacious interventions that target young drivers’ inexperience directly to complement the structure put in place by GDL.

*Enhancing the Intermediate Period of GDL*: Most behavioural interventions directed toward newly licensed teens and their parents are intended to decrease novice drivers’ exposure to the driving environment, especially in conditions known to increase crash risk (e.g., driving at night, with friends) via codifying house rules or increasing parental surveillance using in-vehicle data recorders (IVDRs). Parent-teen agreements (or contracts) have demonstrated promising, but inconsistent effects on reducing teens’ risky driving behaviour and no effect on crashes [4–6]. Many interventions designed to increase parent limit-setting do not offer guidance to parents on best-practices related to parent-adolescent communication (e.g., active listening, respect for the teen’s autonomy), which can result in some parents rejecting the agreements as too business-like, while others embrace the clear contractual boundaries and push them (i.e., “my house my rules”) without soliciting input from their teen. This can diminish the opportunity for the teen to “buy-in” to the idea of limit-setting [7]. Similarly, IVDRs can provide parents with real-time or historical data about adolescents’ driving behaviours, which appears to be a promising strategy for encouraging safe driving [8] However, adolescents view IVDRs as intrusive, lessening their uptake in real-world settings [9, 10]. New solutions are needed that capitalize on the benefit of a strong mutually supportive parent-adolescent relationship, [11, 12] as well as recognize adolescents’ growing need for independence [13].

*Challenges Intervening with Parents and Teens to Promote Teen Driver Safety across GDL*: There continues to be a lack of effective behavioural interventions that directly target young drivers’ inexperience. Young drivers commonly exit the learner period still making considerable safety-relevant errors [14]. Emerging evidence suggests that parenting practices associated with transitioning teens to licensure are a reflection of both a continuation of parenting patterns from earlier developmental periods and anticipation of a new specific challenge salient to mid-adolescence [15]. However, there is very little longitudinal behavioural research on this topic [16]. Collectively, the limited body of research suggests that involved parent supervisors and an emotionally supportive supervisory experience can benefit teen drivers [11]; yet, there is still a strong degree of heterogeneity in how supervision is experienced by families, as well as a lack of experimental research mechanistically connecting parent-teen interactions during the learner phase to entry into the intermediate period of GDL and to safety outcomes. An expert panel convened by the National Highway Traffic Safety Administration (NHTSA) in 2012 concluded that: *“Greater parental involvement may help [reduce crashes] and should be an integral part of GDL and of the overall driver education* process. *Currently, there is no formal preparation for parents for this demanding role”.* [17].

*Need for comprehensive intervention*: The majority of intervention programs and basic research on parent-teen relationship factors concern *newly* licensed teens and their parents [5, 18]. Recently, success in improving specific driving skills (e.g., scanning for hazards) has been consistently demonstrated in applied laboratory settings, and there is emerging evidence to support a meaningful effect on safety as measured by reductions in crash risk [19–21]. Evaluations of driver training programs, primarily examining the contribution of training towards vehicle handling, and studies examining the quantity of practice (e.g., number of supervised practice hours) have both found inconsistent safety outcomes for teens [22–26]. Importantly, many prior evaluations of training programs directed to learner teens and parents have had methodological problems (e.g., lack of random assignment), and the programs themselves have been criticized for a lack of evidence base (e.g., poor correspondence to the task demands of independent driving) and poor engagement [27, 28].

In the current protocol, we evaluate a comprehensive parent-teen intervention, The Drivingly Program, which overcomes these issues. Drivingly is based on strong preliminary data and targets the family unit - as opposed to targeting parents and teens separately. This approach makes the best use of limited resources and is consistent with research indicating that a comprehensive intervention has the strongest potential to reduce MVCs [29].

## Methods

We propose a randomized controlled trial with 1,200 parent-teen dyads to evaluate our central hypothesis, which is that Drivingly can reduce adolescent drivers’ risk for a MVC during the first 12 months of licensure (Hypothesis 1). Our secondary focus is to evaluate Drivingly’s conceptual model (Hypothesis 2 and 3).

Hypothesis 2: Drivingly will:

- improve practice driving (e.g. quantity, diversity, challenge).
- increase the frequency of parent-teen communication about traffic safety driving topics (e.g., GDL).
- increase adolescent drivers’ self-regulation (i.e., self-restriction of driving in conditions that are deemed more challenging and dangerous).
- increase parents’ limit-setting on post-license driving (e.g., passenger restrictions).
- reduce adolescents’ risky driving styles (e.g., angry, high-velocity) and increase safe driving styles (e.g., patient, cautious); and.

Hypothesis 3: Parent-teen (P-T) communication, practice driving, limit-setting, driving style, and self-regulation will mediate the effect of Drivingly on MVC risk.

### Description of Drivingly

The Drivingly Program is based on the Phase Transition Framework for reducing crash risk [30, 31]. It is a multicomponent crash prevention program combining effective interventions 32–35] into a comprehensive program targeting both parents and teens as they move through the licensure process. Drivingly, which spans the learner and junior license periods, consists of two semi-structured health coaching parent sessions, a web-based psychoeducational curriculum with a hazard anticipation and attention training component (e.g., Driver-ZED), and an on-road driver assessment (ODA) and feedback pathway for teen drivers. We review each of these components below.

*Parent-Sessions and Psychoeducational learning-to-drive Curriculum*: Parents receive two, brief, psychoeducational sessions with a trained health coach. The first parent session is an orientation to the program and the learner period, and the second parent session occurs while the teen is taking their ODA and is focused on readiness to drive and keeping teens safe after licensure. These sessions are delivered via a systematic health coach training process and training manual which includes a script to ensure consistency in the delivery of key messages. In complement to these sessions, parents and teens are supported with a web-based psychoeducational curriculum tailored to each stage of GDL and provided with sample conversation starters and activities to clarify goals and expectation for safe driving. The web-based psychoeducational curriculum contains 16 lessons for parents and 13 for teens, plus an online logbook. Structured using the Health Belief Model, 36] the curriculum entails an overview of risk factors for adolescents’ MVCs, the importance of the parental role as practice supervisor and risk-reducer, strategies for overcoming barriers to engagement, and reviews Pennsylvania’s GDL programme. The curriculum consists of two parts. Part 1 is aligned with the permit period and focuses on how to achieve high quality practice driving. There is also a complete practice driving curriculum based on the Drivingly **C**ommunicate **A**nticipate **R**egulate (CAR) Zone system. CAR scaffolds parents’ supervision by clearly defining driving zone specific targets for teens to practice in the domains of communication, anticipation and regulation (e.g., Communicate – Commercial Zone: Look and listen for other drivers and pedestrians trying to communicate with you). Part 2 is aligned with the restricted (or intermediate) license period and scaffolds parents’ involvement during the first year of licensure focusing on parental enforcement of GDL and planning for difficult social situations (e.g., impaired driving).

**Fig. 1:**
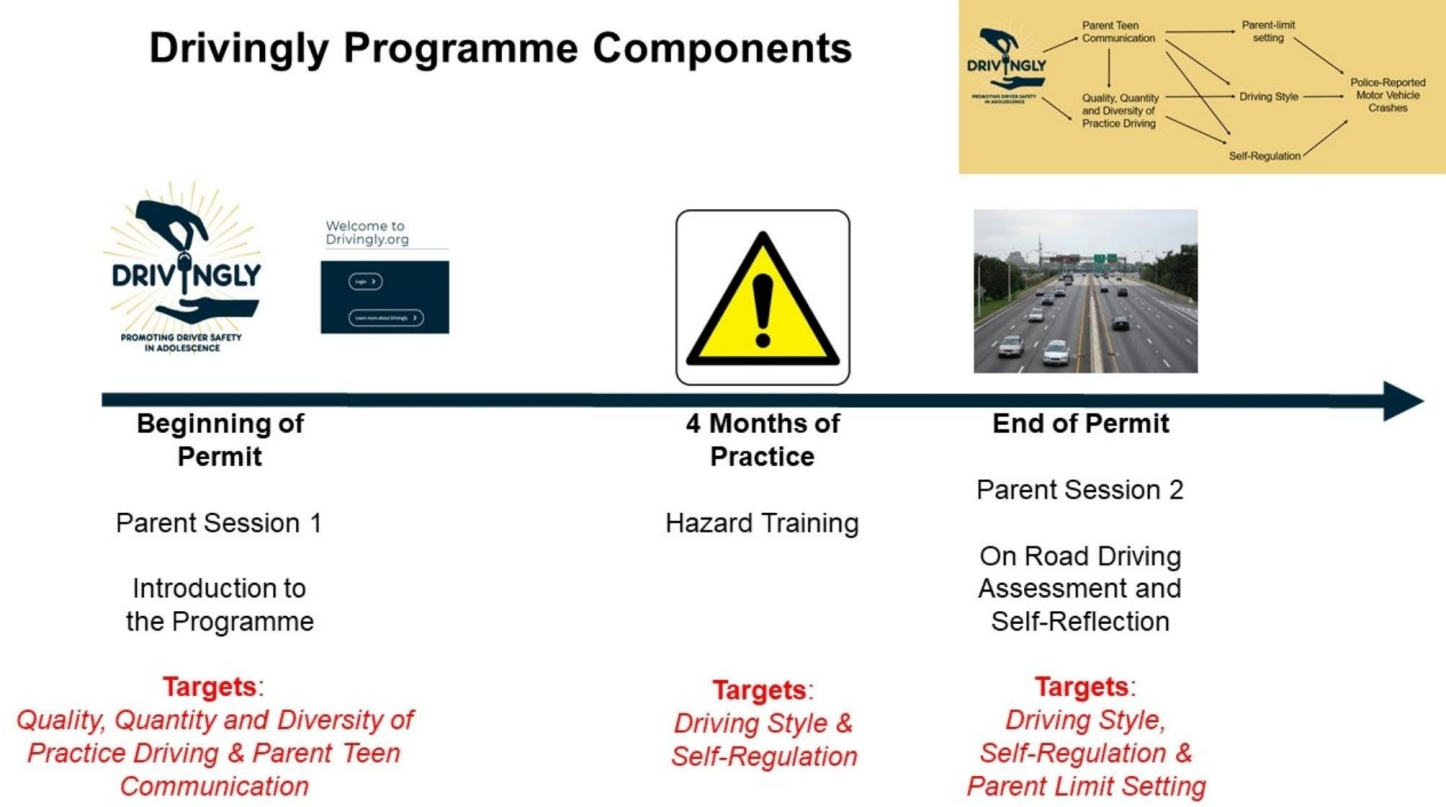
Drivingly Conceptual Model

Prior to commencing the trial, a development phase was undertaken which consisted of four distinct components: (1) refinement of the scientific theory of change, which included the program theory of change (Fig. 1) and implementation plan; (2) content review and development for scalability (and pre-planning for sustainability), including development of online learning modules [https://drivingly.org/], finalization of a tailored on-road assessment and feedback pathway, and intervention administrator training manuals and processes; (3) mixed-method testing of website content for feasibility and acceptability with parents and teens (n = 13, [10 parents and 3 teens]), which included questionnaires on variables of interest from the open and closed-ended responses, as well as short interviews which were thematically analysed; and (4) expert review and feedback from driver educators concerning the on-road driver assessment and feedback methodology. Website feasibility and usability testing indicated high levels of both dimensions across parents, with lower levels among teens. Thematic analysis directed final changes to the web-based component of the program. Qualitative analysis resulted in three themes indicating ways the website could be improved. These included: Theme (1) More interactive activities for teens. Theme (2) More videos for parents and teens. Theme (3) Make information easier to consume on mobile devices. Driver educator feedback on the on-road assessment and feedback session was constructive, indicating feasibility and support. These changes were implemented prior to commencing the trial. A pilot test of a subset of these components (health coaching sessions and a paper version of the parent version of the psychoeducational curriculum) yielded evidence for improved parent-teen communication across a wide range of transportation safety topics [32].

*On-Road Driver Assessment and Feedback Pathway*: Teens receive an on-road driver assessment (ODA) administered by a certified driver instructor in a dual control vehicle in live traffic followed by an individualized feedback session among the instructor, parent and teen. The ODA exposes teens to challenges inherent in key driving environments (e.g., highway, rural areas, commercial roads) and is administered with minimal instruction and guidance by the evaluator, placing the teen fully “in the driver’s seat” mechanically and cognitively. In prior research, when deployed as an outcome assessment, the ODA has been shown to decrease risk for police-reported motor vehicle crashes by half [33]. It has been adapted into an intervention for the purpose of Drivingly. Although the ODA is an interventional component we collect the following data: the start and end time of the assessment, weather conditions, any changes to the route by the evaluator, participant or environmental factors (weather, traffic, road etc.), the number of driving hours the teen has, whether the teen is licensed already, the teen’s written feedback on how they think they performed, the evaluator’s constructive feedback to the teen, and brief notes on any critical driving errors made by the teen in each of the six driving zones (parking lot, residential zone, commercial zone, one and two-lane roadways, rural zone and highway zone). An in-vehicle recording device is used to record all drives; recordings are used for fidelity and quality assurance.

*Drivingly implementation Fidelity and Quality Assurance*: Fidelity checks and quality assurance (QA) will be completed for the first parent session and the ODAs. For the parent sessions, the study team administering the session will fill out a health coach fidelity checklist. Monthly review of the fidelity checklist and ensure that recordings of the parent session are stored on the study’s secure server. A second fidelity check on recorded parent sessions will be conducted by reviewing sampled videos along with the completed health coach fidelity checklist; feedback will be shared with health coaches to ensure minimal deviation from the health coaching manual. For the ODAs, the study team will complete monthly reviews of randomly selected video recordings of the assessment. These will be chosen from a list of all ODAs completed in the previous month with recorded parent sessions. Fidelity checks will be completed by reviewing the recordings of the ODAs along with the evaluation and feedback forms completed by the evaluator during the assessment. Notes of the fidelity checks will then be shared with evaluators to ensure adherence to the protocol. All paperwork completed by assessors and data entry into REDCap will be reviewed weekly by study team members.

### Participants

Eligible participants will include teenagers aged 16-17.33 years age at the time of enrolment, who will have their learner’s permit (Pennsylvania), intend to hold their permit for at least 6 months, have practiced no more than 10 h, and have access to the internet, a practice vehicle, and at least one parent/caregiver for each teen. Parents must be at least 18 years-old, have internet access, and be a licensed driver. Exclusion criteria include non-fluency in written or spoken English, having a sibling enrolled in the study, enrolled in other teen driving studies, and lack of internet and lack of a practice vehicle. Teens with self-reported or parent reported (of teen’s) visual, medical, or physical impairments that would require a handicap placard or assistive device to drive, or teens with pervasive developmental delays are ineligible to participate.

### Recruitment procedures

Participants will be recruited through a variety of strategies including mailings, emails, flyers, presentations in the community, databases for clinical trials (i.e., ResearchMatch ClinicalTrials.gov, etc.), schools and community organizations that give permission, word of mouth, social media postings and advertisements. We will work with the CHOP Recruitment Enhancement Core (REC) and the Pediatric Research Consortium (PeRC) using emails, letters, social media postings (CHOP’s Facebook or Instagram).

Interested people will contact the study team and be screened by phone for eligibility by a member of the study team. The study team will either schedule or re-contact interested participants for consent and assent. The study team will send parents an electronic copy of the consent (through REDCap) and schedule a zoom call with the parent and teen to verbally review the form; parents of participants will review and record written consent electronically in REDCap (via the e-Consent framework). With the REDCap-based e-Consent framework, signatures (typed name and electronic signature via mouse) will be obtained electronically. Parents will be asked to provide their child’s name and email. The REDCap e-consent framework is a secure, web-based, HIPAA-compliant, data collection platform with a user management system allowing project owners to grant and control varying levels of access to data collection instruments and data (e.g. read only, de-identified-only data views) for other users. Upon completion of the consent encounter, participants will be provided with their signed copy of the consent document by email, as well as the option to download a blank form for their records. Teens will then provide written assent via REDCap’s e-consent. In assent, participants will be asked to provide their signature, first and last name, date of birth, email, and the date that they are signing the form. After assent, participants will be emailed or able to download a copy of their signed form. If a participant turns 18 after enrolment, they will be re-consented again using the REDCap e-Consent framework. After dyads have documented parental consent, parental permission, and provided teen assent (baseline) and eligibility is confirmed, dyads will be randomized using a table of random numbers and start survey procedures.

### Compensation

Parent and teen participants will be compensated separately via study issued ClinCards. Teens in the control group will be compensated a total of $245 distributed across 6 surveys. They will receive $25 for completing the baseline survey, $20 for each of the 3 month post-baseline and post-licensure surveys, and $60 for each of the 3, 6, and 12 month post-licensure surveys. Teens in the intervention group will also receive $245 for completing all 6 surveys, as well as an additional $100 for completing the on-road driver assessment. Parent participants in both groups will be compensated a total of $65 for completing 3 surveys. They will receive $25 for the baseline survey and $20 for the 3 month post-baseline and post-licensure surveys.

### Study measurements

#### Crash

We will request crash records from the Pennsylvania (PA) Department of Transportation (DOT) for the teen participants enrolled in the study following previously validated procedures [33]. Crash reports will be requested for all licensed teen participants and sent to the study team via a secure FTP.

#### Use of Drivingly Online materials

Data on parent and teen online engagement will be captured using passive web-tracking enabled on the Drivingly web platform and tagged to log-in credentials. Thus, we will be able to evaluate engagement with the various components of the psychoeducational curriculum. This information will be used for quality improvement and for any “as-treated” analyses.

#### Surveys

Survey collection from teens will occur at baseline, 3 months post-baseline, licensure, 3 months post-licensure, 6 months post-licensure, and 12months post-licensure. Survey collection from parents will occur at baseline, 3 months post baseline, and at teen licensure. Survey collection will be done remotely, through REDCap. Missed survey responses may be followed up by phone, text, or email with the study team. Additionally, dyads will be asked to record/log their practice driving for 5–6 months post-baseline and prior to obtaining their driver’s license via a web-based e-learning platform (via the Drivingly WordPress platform). See Supplementary Table 1 for a summary of survey measures mapped to the Drivingly Conceptual Model. In addition, we measured driving exposure two ways at baseline, 3, 6, 9, 12 and 18 months with The Situational Driving Frequency Scale [37] to assess driving frequency in 14 situations using 5-point scale ranging from Never (0) to Very Often (4 to 7 days per week); total score 0–56 (teen report; parent report at Baseline only) and hours of driving per week was assessed using a write-in of hours driven Monday-Friday; Saturday and Sunday at (teen and parent report). Sociodemographic data were collected at baseline and included teen and parent gender, age, race and ethnicity, and parent’s relationship to teen, educational attainment, and current relationship status. Additional individual difference variables were collected but are not the focus of the main trial (e.g., sensation seeking) and are thus not reported here.

### Statistical plan

With an initial sample size of 600 Drivingly dyads and 600 controls assuming 10% dropout in each group, a 1-year post-licensure crash risk in the unexposed group of 15%, and significance level (α) of 0.05, we will have 90% power to detect a hazard ratio (HR) of 0.59 for the first year of licensure. Alternatively, assuming a first-year control group crash rate of 20% the detectable HR is 0.65 for the first year of licensure; assuming a 25% first-year crash rate, the detectable HR is 0.69 [38]. Given our prior observed reduction of the ODA on crash risk of 53% (adjusted HR 0.47), these detectable HRs are conservative. Specifically, if the true HR is 0.47, we will have 99.4% power if the control group crash rate is 15%.

Risk ratios comparing the risk of any MVC involvement between Drivingly and Control will be estimated using modified Poisson regression [39]. Time-to-event (survival) analysis will be conducted to examine time to first MVC (from time of enrolment and from intermediate licensure) as the primary dependent variables of interest. We will create Kaplan-Meier curves to graphically depict the probability of these outcomes and compare the distributions of the two groups (Drivingly vs. Control) using the log-rank test. We will use a Cox proportional hazards (PH) model to estimate unadjusted hazard ratios, using an exact method for handling tied event times. To determine if there is effect modification by gender, we will use a PH model with a multiplicative interaction between gender and group (Drivingly vs. Control) and assess significance at the 0.10 level. We will report gender-specific hazard ratios between the Drivingly and Control groups because male drivers are at greater risk for MVCs than female drivers [40, 41].

To evaluate Drivingly’s conceptual model (Fig. 1) we will use marginal structural models with VanderWeele’s decomposition formulas for mediation analyses [42] This counterfactual approach to mediation analysis allows for the decomposition of total effects into direct and indirect components so we can estimate the proportion of the total effect of Drivingly on MVC risk mediated by each hypothesized process variable [42].

If there is substantial item-level missingness in the survey data (e.g., in excess of 5%), we will assess whether non-response bias may be present; however, if we find such differences, we will use multiple imputation with chained equations as suggested by prior research under the relatively mild assumption of missingness at random [43].

### Data Safety Monitoring Board

The MPIs and select study team members will meet with a Data and Safety Monitoring Board (DSMB) biannually to ensure appropriate study progress, conduct, and safety. The DSMB is comprised of a Chair and two board members. Meeting minutes will be taken and drafted into a letter signed by the Chair. After each biannual meeting, the study teem will update procedures per the DSMB’s recommendation.

### Ethical review

This protocol was reviewed and approved by the IRB the University of Pennsylvania. Enrolment began on August 18, 2021 and is ongoing at the time of submission.

## Discussion

Although recruitment was delayed by the COVID-19 pandemic, enrollment has been robust. A number of infection control measures were taken by the study team, including masking and covid-screening protocols per the University of Pennsylvania’s COVID-19 and local guidelines where the ODAs take place, sanitation of training vehicles between each ODA, and air filtration systems in all communal spaces, but these have not negatively affected operations. The target completion date for the study is June 2025 and recruitment is on-going at the time of submission of this article for publication.

Young drivers’ crash risk is highly heterogeneous, changes non-linearly and non-incrementally, and the variability of the transportation environment in conjunction with its interconnected (i.e., systems) features make individual-level crash risk hard to predict [31]. Thus, a flexible, multicomponent, and sustained set of cohesive interventions stands the best chance to meaningfully reduce crash risk for the largest number of young drivers. Results from the main trial, which commenced in the summer of 2021, will help inform clear strategies to reduce motor vehicle crashes.

### Electronic supplementary material

Below is the link to the electronic supplementary material.

Supplementary Material 1

Supplementary Material 2

## Data Availability

The datasets used and/or analysed during the current study will be available from the corresponding author on reasonable request and de-identified data will be made available via a persistent link included with each publication.

## Abbreviations

MVC: Motor Vehicle Crash(es)
PA: DOT Pennsylvania Department of Transportation
