# Supplementary material for: Promoting transportation safety in adolescence: the drivingly randomized controlled trial
Source: BMC Public Health. 2023 Oct 17;23:2020. doi: 10.1186/s12889-023-16801-6 (PMC10580546; doi:10.1186/s12889-023-16801-6)
Supplement: Supplementary file 1 — Supplementary Material 1 [file 12889_2023_16801_MOESM1_ESM.docx]

| **Supplementary Table 1. Survey Assessments of Drivingly Conceptual Model** | | | |
| --- | --- | --- | --- |
| **Construct** | **Description of Measure** | **Timing** (months from baseline) | **Reporter** |
| **“Quality, Quantity and Diversity of Practice Driving” is measured using three constructs and three measures capturing behind-the-wheel supervision in different driving areas, time in driving in different driving environments and focus on specific skills.** | | | |
| Practice Diversity and Quality | Diversity and quality of parent supervised practice is assessed using 9 items with three parts adapted from a prior measure of Practice Diversity^1^. Part 1: yes/no if practice occurred in specific environments (e.g. highway), Part 2 selection of the number of hours practiced using closed-ended response format, Part 3 yes/no if specific skills were practiced in each environment. | Baseline, 3, and 6 | Parent, Teen |
| Parent Engagement | Parental Engagement In Practice Supervision Scale (PEPSS)^1^  Parent practice engagement is measured by 10 items on a five-point scale ranging from “never” (1) to “always” (5) (.e.g., I decided what driving skills I wanted my teen to practice); average total score 1-5. | Baseline | Parent |
| **“Parent-Teen Communication and Support” is measured by two constructs, social support and content-specific communication about driver safety topics.** | | | |
| Perceived Support | Practice Driving Support Scale (PDSS)^2^  Parents and teens report on perceived support received during the practice driving process using 4 items (e.g., Overall, I felt my parent supported me in the practice driving process) using 5-point scale Never (1) to Always 5); average total score 1-5. Teens report on parents and parents report on teens. | Baseline, 3 and 6 | Parent, Teen |
| Parent-teen communication on driver safety topics | Communication on driver safety topics scale (CDSTS)^3^  Parents and teens report the frequency or discussion about 12 teem driver safety topics (e.g., the kinds of risky driving situations that might come up in teen’s friend group) using one of the following response choices: “never talked about” (0) to “talked about a lot, about 5 times or more” (3); average total score 1-3 | 6 | Parent, Teen |
| **“Parent-Limit Setting” and Health Belief Model Constructs are measured by bespoke surveys tailored to the Drivingly trial in conjunction with a bespoke GDL knowledge scale** | | | |
| Health Belief Model Survey | This survey consists of 34 items mapped to Health Belief Model Constructs: Perceived susceptibility (3 items), crash risk knowledge (12 items) and injury risk (3 items) using a 7 point scale from Significantly Decreases (1) to Significantly Increases (7); benefits (8 items) and barriers (8) were assessed using a 7-point scale Not at all True (1) to Very True of Me (7) (e.g., It was hard to find time to practice.). | 6 | Parent |
| Parent Limit Setting | 12 items on parents’ imposition of driving limits; item content informed by the Checkpoints Trial^4^ (e.g., a rule that you cannot drive with more than one friend in the car at a time?) were assessed using a 5-point scale from Never (1) to Always (5); average total score 1-5. | 6, 9,12, and 18 | Teen |
| **Driving Avoidance/self-regulation and Driving Style** | | | |
| Driving Avoidance | Driving Avoidance is assessed using the Driving Habits Questionnaire which consists of 9 items answered on a 5 point scale, Do you avoid driving at night? (1) Never to (5) Always. | 9,12, and 18 | Teen |
| Driving Style, Teen | The Driver Self Rating Scale.^5,6^ 19 items assessed on a bi-polar 7-point scale (e.g. Safe (1) – Unsafe (7)). | 9,12, and 18 | Teen |
| Perceptions of Safety, Expertise, Confidence | Respondents answered three questions about safety, expertise and confidence using 5 point scales: ^7^ Compared to other teens with as much driving experience, I would rate my skills as…”much below average (1) to much above average (5); I would rate myself as a beginner driver (1) to expert driver (5); and Compared to other drivers in general, I would rate myself as a…(1) very unsafe to very safe (5). | 9,12, and 18 | Teen |
| **Adolescents’ post-license risky driving behaviour is measured with three constructs focusing on risky driving behaviours and errors, GDL violations, citations and crashes** | | | |
| Risky Driving | Risky driving is assessed using two measures, The Driver Behaviour Questionnaire^8^ consisting of 28 risky driving items assessing aberrant driving behaviour (Lapses, Errors, Violations and Aggressive Violations) using a 6 point scale Never (0) to Nearly all the time (5) (e.g., I ignore speed limits late at night or early in the morning) and a 12-item assessment of the frequency of engagement in in higher-risk scenarios (e.g., How often do you drive while under the influence of prescription drugs taken for recreational use?) using a 5 point scale (1) Never to 5 (Almost all trips). | 9, 12, and 18 | Teen |
| GDL Violations | Teens reported respond to 6 items developed for this study mapped to compliance with Pennsylvania GDL regulations (e.g., passenger limits) on a 6 point scale Never (1) to Nearly all the Time (6); average total score 1-6 | 6, 9, 12, and 18 | Teen |
| Citations and Crashes | Teens self-report violations in which they received a citation and crashes (note the main endpoint is police reported motor vehicle crashes; however, we had teens self-report as well which can facilitate identification of out of state crashes). | 9,12, and 18 | Teen |

References

1. Mirman JH, Albert WD, Curry AE, Winston FK, Fisher Thiel MC, Durbin DR. TeenDrivingPlan Effectiveness: The Effect of Quantity and Diversity of Supervised Practice on Teens’ Driving Performance. *J Adolesc Health*. 2014;55(5):620-626. doi:10.1016/j.jadohealth.2014.04.010

2. Mirman JH, Curry AE, Wang W, Fisher Thiel MC, Durbin DR. It takes two: A brief report examining mutual support between parents and teens learning to drive. *Accid Anal Prev*. 2014;69:23-29. doi:10.1016/j.aap.2013.10.006

3. Mirman JH, Goodman ES, Friedrich E, Ford CA. Talking with teens about traffic safety: Initial feasibility, acceptability, and efficacy of a parent-targeted intervention for primary care settings. *J Safety Res*. 2018;66:113-120. doi:10.1016/j.jsr.2018.06.008

4. Simons-Morton BG, Hartos JL, Leaf WA, Preusser DF. Persistence of Effects of the Checkpoints Program on Parental Restrictions of Teen Driving Privileges. *Am J Public Health*. 2005;95(3):447-452. doi:10.2105/AJPH.2003.023127

5. Guppy A, Wilson P, Perry J. Driving attitudes and driving experience. In: *Driving Behaviour in a Social Context*. ; 1990:181-189.

6. Wells P, Tong S, Sexton B, Grayson G, Jones E. *Cohort II: A Study of Learner and New Drivers: Volume 1: Main Report*.; 2008. Accessed September 1, 2023. https://trid.trb.org/view/1153843

7. Mirman JH, Curry AE, Winston FK, et al. Parental influence on driver licensure in adolescence: A randomized controlled trial. *Health Psychol*. 2017;36(3):245-254. doi:10.1037/hea0000444

8. Af Wåhlberg A, Dorn L, Kline T. The Manchester Driver Behaviour Questionnaire as a predictor of road traffic accidents. *Theor Issues Ergon Sci*. 2011;12(1):66-86. doi:10.1080/14639220903023376
